# Di-4-pyridyl disulfide–isophthalic acid (1/1)

**DOI:** 10.1107/S1600536809013397

**Published:** 2009-04-18

**Authors:** Dan-Jun Wang, Jun-Ming Zhao, Jie Zhang, Jian-Li Lin

**Affiliations:** a State Key Laboratory Base of Novel Functional Materials and Preparation Science, Faculty of Materials Science and Chemical Engineering, Institute of Solid Materials Chemistry, Ningbo University, Ningbo, Zhejiang 315211, People’s Republic of China

## Abstract

In the title 1:1 cocrystal, C_10_H_8_N_2_S_2_·C_8_H_6_O_4_, the asymmetric unit contains an isophthalic acid mol­ecule and a 4,4′-dipyridyl disulfide mol­ecule. The two carboxyl groups of isophthalic acid inter­act with neighbouring 4,4′-dipyridyl disulfide mol­ecules through O—H⋯N hydrogen bonds, forming a one-dimensional zigzag chain. Neighbouring chains are linked to each other *via* π–π stacking inter­actions between the pyridyl rings of adjacent 4,4′-dipyridyl disulfide mol­ecules [centroid-centroid distance = 3.7346 (6) Å], resulting in a layered motif. The dihedral angle between pyridine rings of 84.13 (7)° and the C—S—S—C torsion angle of 91.95 (1)° confirm the *gauche* conformation of 4,4′-dipyridyl disulfide.

## Related literature

For ligands with two 4-pyridyl donors, see: Biradha *et al.* (2006 ▶); Sun *et al.* (2006 ▶); He *et al.* (2008 ▶); Suen *et al.* (2005 ▶). For related structures, see: Ranjbar *et al.* (2007 ▶).
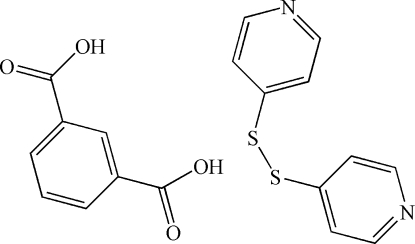


## Experimental

### 

#### Crystal data

C_10_H_8_N_2_S_2_·C_8_H_6_O_4_
*M* *_r_* = 386.43
Monoclinic, 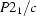

*a* = 5.9616 (12) Å
*b* = 10.024 (2) Å
*c* = 29.797 (6) Å
β = 93.71 (3)°
*V* = 1776.9 (6) Å^3^
*Z* = 4
Mo *K*α radiation
μ = 0.33 mm^−1^
*T* = 295 K
0.29 × 0.20 × 0.11 mm

#### Data collection

Rigaku R-AXIS RAPID diffractometer
Absorption correction: multi-scan (*ABSCOR*; Higashi, 1995 ▶) *T*_min_ = 0.920, *T*_max_ = 0.964
16923 measured reflections
4039 independent reflections
2330 reflections with *I* > 2σ(*I*)
*R*_int_ = 0.048

#### Refinement

*R*[*F* ^2^ > 2σ(*F* ^2^)] = 0.043
*wR*(*F* ^2^) = 0.135
*S* = 1.08
4039 reflections
235 parameters
H-atom parameters constrained
Δρ_max_ = 0.30 e Å^−3^
Δρ_min_ = −0.41 e Å^−3^

### 

Data collection: *RAPID-AUTO* (Rigaku, 1998 ▶); cell refinement: *RAPID-AUTO*; data reduction: *CrystalStructure* (Rigaku/MSC, 2004 ▶); program(s) used to solve structure: *SHELXS97* (Sheldrick, 2008 ▶); program(s) used to refine structure: *SHELXL97* (Sheldrick, 2008 ▶); molecular graphics: *SHELXTL* (Sheldrick, 2008 ▶); software used to prepare material for publication: *SHELXL97*.

## Supplementary Material

Crystal structure: contains datablocks global, I. DOI: 10.1107/S1600536809013397/pv2150sup1.cif

Structure factors: contains datablocks I. DOI: 10.1107/S1600536809013397/pv2150Isup2.hkl

Additional supplementary materials:  crystallographic information; 3D view; checkCIF report

## Figures and Tables

**Table 1:** Hydrogen-bond geometry (Å, °)

*D*—H⋯*A*	*D*—H	H⋯*A*	*D*⋯*A*	*D*—H⋯*A*
O2—H2*C*⋯N1^i^	0.99	1.64	2.629 (3)	175
O4—H4*C*⋯N2^ii^	0.81	1.85	2.651 (3)	176

Symmetry codes: (i) 
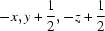
; (ii) 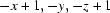.

**Fig. 1.**
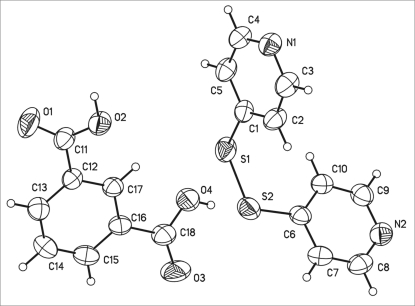
A view of the molecular structure of the title cocrystal, displacement ellipsoids are drawn at the 45% probability level.

**Fig. 2.**
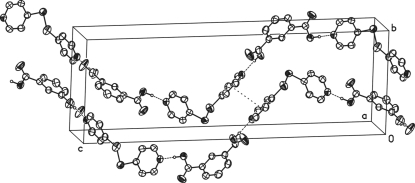
The crystal packing diagram, showing the π—π stacking and hydrogen bonds as dash lines.

**Fig. 3.**
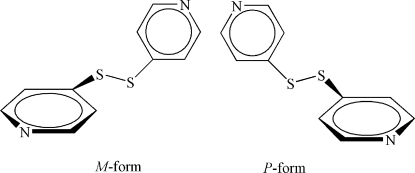
The M- and P-enantiomers of di-4-pyridyl disulfide.

## supplementary crystallographic information

### Comment

The ligands having two 4-pyridyl donors, *e.g.*, 4,4'-bipyridine (Biradha *et al.*,2006), 1,2-bis(4-pyridyl)ethane (Sun *et al.*, 2006) and 1,3-bis(4-pyridyl)-propane (He *et al.*, 2008) have been intensively employed for the construction of coordination polymers. Compared with the above examples, di-4-pyridyl disulfide is seldom used for research. It shows a twisted strcture, with a C—S—S—C torsion angle of approximately 90°. More importantly, the ligand has axial chirality, which generates *M-* and *P-* enantiomers as shown in Fig. 3. It means that the use of this ligand possibly can produce the complex with a non-centrosymmetric space group (Suen *et al.*, 2005). As we know, some special properties, *e.g.*, triboluminescence, second harmonic generation and ferroelectricity are only found in these materials. For this consideration, we mixed this ligand and carboxylate ligand hoping to gain coordination polymer with special properties. However, a crystal suitable for X-ray diffraction was obtained during the synthesis unexpectedly. In this paper we report the crstal structure of the title cocrystal.

The asymmetric unit of the title cocrystal consisits of one isophthalic acid molecule and one *P-* form di-4-pyridyl disulfide molecule (Fig. 1). The two carboxylic groups of the isophthalic acid are hydrogen bonded with the corresponding di-4-pyridyl disulfide molecules (O2—H2C···N1^i^ and O4—H4C···N2^ii^ (Table 1)) generating a one-dimensional zigzag chain along the *c* axis. The neighbouring chains are further linked to each other *via*π—π packing interactions between the pyridyl rings of adjacent di-4-pyridyl disulfide molecules resulting in a two-dimensional layered structure (Fig. 2). The centroid-centroid distance is 3.7346 (6) Å, the C—S—S—C torsion angle is 91.95 (1)°, and the pyridyl ring planes form a dihedral angle of 84.13 (7)°. The crystal structures of closesly related cocrystals have been reported (Ranjbar *et al.*, 2007).

### Experimental

Dropwise addition of Na_2_CO_3_ (0.5 ml 1.0 *M*) to an aqueous solution of Zn(NO_3_)_2_.6H_2_O (0.0808 g, 0.25 mmol)in 4 ml H_2_O produced white precipitate, which was then centrifuged and washed with distilled water six times. The collected precipitate was subsequently moved to a stirred suspension of isophthalic acid ( 0.0817 g, 0.5 mmol) in a mixed solvent composed of EtOH (10 ml) and H_2_O (20 ml), and further stirred at 353 K for 1 h, followed by the addition of an ethanolic solution of 0.1120 g (0.5 mmol) di-4-pyridyl disulfide in 5 ml EtOH. The resulting mixture was further stirred at 343 K for 30 min and filtered off. Slow evaporation of the colorless filtrate at room temperature for one week gave colorless block crystals (yield: 0.05 g).

### Refinement

H atoms bonded to C atoms were palced in geometrically calculated position and were refined using a riding model, with *U*_iso_(H) = 1.2 *U*_eq_(C). H atoms attached to O atoms were found in a difference Fourier synthesis and were refined using a riding model, with the O—H distances fixed as initially found and with *U*_iso_(H) values set at 1.2 *U*eq(O).

### Crystal data

**Table:**

C_10_H_8_N_2_S_2_·C_8_H_6_O_4_	*F*(000) = 800
*M**_r_* = 386.43	*D*_x_ = 1.445 Mg m^−^^3^
Monoclinic, *P*2_1_/*c*	Mo *K*α radiation, λ = 0.71073 Å
Hall symbol: -P 2ybc	Cell parameters from 16923 reflections
*a* = 5.9616 (12) Å	θ = 3.4–27.4°
*b* = 10.024 (2) Å	µ = 0.33 mm^−^^1^
*c* = 29.797 (6) Å	*T* = 295 K
β = 93.71 (3)°	Platelet, colorless
*V* = 1776.9 (6) Å^3^	0.29 × 0.20 × 0.11 mm
*Z* = 4	

### Data collection

**Table:**

Rigaku R-AXIS RAPID diffractometer	4039 independent reflections
Radiation source: fine-focus sealed tube	2330 reflections with *I* > 2σ(*I*)
graphite	*R*_int_ = 0.048
Detector resolution: 0 pixels mm^-1^	θ_max_ = 27.4°, θ_min_ = 3.4°
ω scans	*h* = −7→7
Absorption correction: multi-scan (*ABSCOR*; Higashi, 1995)	*k* = −12→12
*T*_min_ = 0.920, *T*_max_ = 0.964	*l* = −38→38
16923 measured reflections	

### Refinement

**Table:**

Refinement on *F*^2^	Primary atom site location: structure-invariant direct methods
Least-squares matrix: full	Secondary atom site location: difference Fourier map
*R*[*F*^2^ > 2σ(*F*^2^)] = 0.043	Hydrogen site location: inferred from neighbouring sites
*wR*(*F*^2^) = 0.135	H-atom parameters constrained
*S* = 1.08	*w* = 1/[σ^2^(*F*_o_^2^) + (0.0469*P*)^2^ + 0.8478*P*] where *P* = (*F*_o_^2^ + 2*F*_c_^2^)/3
4039 reflections	(Δ/σ)_max_ < 0.001
235 parameters	Δρ_max_ = 0.30 e Å^−^^3^
0 restraints	Δρ_min_ = −0.41 e Å^−^^3^

### Special details

**Table:**

Geometry. All e.s.d.'s (except the e.s.d. in the dihedral angle between two l.s. planes) are estimated using the full covariance matrix. The cell e.s.d.'s are taken into account individually in the estimation of e.s.d.'s in distances, angles and torsion angles; correlations between e.s.d.'s in cell parameters are only used when they are defined by crystal symmetry. An approximate (isotropic) treatment of cell e.s.d.'s is used for estimating e.s.d.'s involving l.s. planes.
Refinement. Refinement of *F*^2^ against ALL reflections. The weighted *R*-factor *wR* and goodness of fit *S* are based on *F*^2^, conventional *R*-factors *R* are based on *F*, with *F* set to zero for negative *F*^2^. The threshold expression of *F*^2^ > σ(*F*^2^) is used only for calculating *R*-factors(gt) *etc*. and is not relevant to the choice of reflections for refinement. *R*-factors based on *F*^2^ are statistically about twice as large as those based on *F*, and *R*- factors based on ALL data will be even larger.

### Fractional atomic coordinates and isotropic or equivalent isotropic displacement parameters (Å^2^)

**Table:**

	*x*	*y*	*z*	*U*_iso_*/*U*_eq_	
C1	0.1566 (4)	0.1783 (3)	0.32831 (8)	0.0472 (6)	
C2	0.1371 (5)	0.0516 (3)	0.31039 (10)	0.0570 (7)	
H2A	0.2419	−0.0142	0.3187	0.068*	
C3	−0.0411 (5)	0.0247 (3)	0.27995 (10)	0.0616 (8)	
H3A	−0.0545	−0.0611	0.2682	0.074*	
N1	−0.1955 (4)	0.1147 (2)	0.26642 (7)	0.0541 (6)	
C4	−0.1742 (5)	0.2369 (3)	0.28378 (9)	0.0542 (7)	
H4A	−0.2806	0.3011	0.2748	0.065*	
C5	−0.0019 (5)	0.2723 (3)	0.31427 (9)	0.0526 (7)	
H5A	0.0079	0.3589	0.3254	0.063*	
S1	0.36292 (14)	0.23223 (8)	0.36981 (3)	0.0629 (2)	
S2	0.59614 (12)	0.08517 (9)	0.37425 (3)	0.0664 (3)	
C6	0.5151 (4)	−0.0234 (3)	0.41697 (8)	0.0467 (6)	
C7	0.6649 (4)	−0.1247 (3)	0.42909 (9)	0.0543 (7)	
H7A	0.7977	−0.1335	0.4146	0.065*	
C8	0.6162 (5)	−0.2124 (3)	0.46265 (10)	0.0588 (8)	
H8A	0.7195	−0.2791	0.4708	0.071*	
N2	0.4262 (4)	−0.2055 (3)	0.48407 (7)	0.0571 (6)	
C9	0.2814 (5)	−0.1082 (3)	0.47191 (9)	0.0558 (7)	
H9A	0.1483	−0.1031	0.4865	0.067*	
C10	0.3170 (4)	−0.0152 (3)	0.43917 (9)	0.0520 (7)	
H10A	0.2119	0.0513	0.4321	0.062*	
O1	0.6473 (5)	0.7527 (3)	0.28620 (9)	0.0996 (9)	
O2	0.5074 (3)	0.5519 (2)	0.29642 (6)	0.0634 (6)	
H2C	0.3967	0.5742	0.2715	0.095*	
C11	0.6474 (5)	0.6489 (3)	0.30626 (9)	0.0528 (7)	
C12	0.8127 (4)	0.6179 (3)	0.34480 (8)	0.0452 (6)	
C13	1.0212 (5)	0.6802 (3)	0.34736 (9)	0.0541 (7)	
H13A	1.0553	0.7426	0.3257	0.065*	
C14	1.1780 (5)	0.6495 (3)	0.38191 (10)	0.0603 (8)	
H14A	1.3179	0.6909	0.3833	0.072*	
C15	1.1292 (4)	0.5580 (3)	0.41451 (9)	0.0574 (8)	
H15A	1.2368	0.5368	0.4374	0.069*	
C16	0.9196 (4)	0.4977 (3)	0.41308 (8)	0.0467 (6)	
C17	0.7617 (4)	0.5277 (3)	0.37792 (8)	0.0451 (6)	
H17A	0.6214	0.4868	0.3767	0.054*	
C18	0.8684 (5)	0.3991 (3)	0.44862 (9)	0.0566 (7)	
O3	1.0115 (4)	0.3410 (3)	0.47123 (9)	0.0996 (9)	
O4	0.6531 (3)	0.3815 (2)	0.45246 (7)	0.0695 (6)	
H4C	0.6275	0.3313	0.4726	0.104*	

### Atomic displacement parameters (Å^2^)

**Table:**

	*U*^11^	*U*^22^	*U*^33^	*U*^12^	*U*^13^	*U*^23^
C1	0.0550 (16)	0.0416 (15)	0.0445 (14)	−0.0047 (12)	0.0007 (12)	0.0048 (12)
C2	0.0646 (18)	0.0438 (17)	0.0606 (17)	0.0055 (14)	−0.0107 (14)	−0.0019 (13)
C3	0.078 (2)	0.0459 (17)	0.0589 (17)	−0.0006 (16)	−0.0134 (16)	−0.0092 (14)
N1	0.0640 (15)	0.0536 (15)	0.0433 (12)	−0.0002 (12)	−0.0078 (11)	0.0003 (11)
C4	0.0624 (17)	0.0485 (17)	0.0509 (15)	0.0066 (14)	−0.0027 (14)	0.0038 (13)
C5	0.0681 (18)	0.0391 (15)	0.0502 (15)	0.0009 (14)	−0.0005 (14)	−0.0019 (12)
S1	0.0717 (5)	0.0503 (5)	0.0638 (5)	−0.0141 (4)	−0.0174 (4)	0.0058 (4)
S2	0.0482 (4)	0.0838 (6)	0.0672 (5)	−0.0068 (4)	0.0036 (3)	0.0218 (4)
C6	0.0386 (13)	0.0557 (17)	0.0447 (13)	−0.0022 (12)	−0.0055 (11)	−0.0001 (12)
C7	0.0433 (15)	0.0660 (19)	0.0527 (16)	0.0059 (14)	−0.0033 (12)	−0.0024 (14)
C8	0.0587 (17)	0.0601 (19)	0.0552 (16)	0.0083 (15)	−0.0144 (14)	0.0001 (15)
N2	0.0612 (15)	0.0606 (16)	0.0477 (13)	−0.0051 (13)	−0.0106 (11)	0.0067 (11)
C9	0.0489 (15)	0.067 (2)	0.0510 (15)	−0.0048 (15)	0.0008 (13)	0.0040 (14)
C10	0.0434 (14)	0.0571 (18)	0.0551 (15)	0.0022 (13)	−0.0007 (12)	0.0064 (14)
O1	0.118 (2)	0.0680 (17)	0.1053 (19)	−0.0274 (15)	−0.0499 (16)	0.0421 (15)
O2	0.0698 (13)	0.0577 (13)	0.0589 (12)	−0.0097 (11)	−0.0244 (10)	0.0090 (10)
C11	0.0612 (17)	0.0465 (17)	0.0495 (15)	−0.0013 (14)	−0.0059 (13)	0.0043 (13)
C12	0.0509 (15)	0.0405 (15)	0.0435 (13)	0.0004 (12)	−0.0026 (12)	−0.0027 (11)
C13	0.0570 (17)	0.0544 (18)	0.0507 (15)	−0.0046 (14)	0.0011 (13)	0.0000 (13)
C14	0.0458 (15)	0.071 (2)	0.0634 (18)	−0.0101 (15)	−0.0001 (14)	−0.0047 (16)
C15	0.0455 (15)	0.072 (2)	0.0526 (16)	0.0031 (15)	−0.0103 (13)	−0.0050 (15)
C16	0.0452 (14)	0.0530 (17)	0.0408 (13)	0.0033 (12)	−0.0054 (11)	−0.0026 (12)
C17	0.0446 (14)	0.0447 (15)	0.0449 (13)	0.0002 (12)	−0.0044 (11)	−0.0011 (12)
C18	0.0541 (16)	0.069 (2)	0.0454 (15)	0.0018 (15)	−0.0095 (13)	0.0063 (14)
O3	0.0644 (14)	0.135 (2)	0.0961 (18)	0.0153 (16)	−0.0159 (13)	0.0616 (18)
O4	0.0580 (12)	0.0864 (16)	0.0624 (12)	−0.0072 (11)	−0.0107 (10)	0.0307 (11)

### Geometric parameters (Å, °)

**Table:**

C1—C5	1.380 (4)	C9—H9A	0.9300
C1—C2	1.380 (4)	C10—H10A	0.9300
C1—S1	1.771 (3)	O1—C11	1.200 (3)
C2—C3	1.378 (4)	O2—C11	1.302 (3)
C2—H2A	0.9300	O2—H2C	0.9857
C3—N1	1.333 (4)	C11—C12	1.497 (4)
C3—H3A	0.9300	C12—C17	1.387 (4)
N1—C4	1.333 (4)	C12—C13	1.388 (4)
C4—C5	1.373 (4)	C13—C14	1.379 (4)
C4—H4A	0.9300	C13—H13A	0.9300
C5—H5A	0.9300	C14—C15	1.381 (4)
S1—S2	2.0248 (13)	C14—H14A	0.9300
S2—C6	1.766 (3)	C15—C16	1.386 (4)
C6—C7	1.385 (4)	C15—H15A	0.9300
C6—C10	1.393 (3)	C16—C17	1.395 (3)
C7—C8	1.376 (4)	C16—C18	1.495 (4)
C7—H7A	0.9300	C17—H17A	0.9300
C8—N2	1.338 (4)	C18—O3	1.203 (3)
C8—H8A	0.9300	C18—O4	1.308 (3)
N2—C9	1.337 (4)	O4—H4C	0.8043
C9—C10	1.376 (4)		
			
C5—C1—C2	118.2 (3)	C10—C9—H9A	118.0
C5—C1—S1	115.7 (2)	C9—C10—C6	118.1 (3)
C2—C1—S1	126.0 (2)	C9—C10—H10A	120.9
C3—C2—C1	118.5 (3)	C6—C10—H10A	120.9
C3—C2—H2A	120.8	C11—O2—H2C	112.9
C1—C2—H2A	120.8	O1—C11—O2	123.7 (3)
N1—C3—C2	123.7 (3)	O1—C11—C12	122.7 (3)
N1—C3—H3A	118.2	O2—C11—C12	113.5 (2)
C2—C3—H3A	118.2	C17—C12—C13	119.4 (2)
C3—N1—C4	117.3 (2)	C17—C12—C11	121.2 (2)
N1—C4—C5	122.8 (3)	C13—C12—C11	119.5 (2)
N1—C4—H4A	118.6	C14—C13—C12	120.1 (3)
C5—C4—H4A	118.6	C14—C13—H13A	119.9
C4—C5—C1	119.5 (3)	C12—C13—H13A	119.9
C4—C5—H5A	120.2	C13—C14—C15	120.6 (3)
C1—C5—H5A	120.2	C13—C14—H14A	119.7
C1—S1—S2	105.44 (10)	C15—C14—H14A	119.7
C6—S2—S1	106.08 (10)	C14—C15—C16	119.9 (3)
C7—C6—C10	118.1 (3)	C14—C15—H15A	120.0
C7—C6—S2	116.0 (2)	C16—C15—H15A	120.0
C10—C6—S2	125.9 (2)	C15—C16—C17	119.5 (3)
C8—C7—C6	119.7 (3)	C15—C16—C18	119.4 (2)
C8—C7—H7A	120.2	C17—C16—C18	121.0 (2)
C6—C7—H7A	120.2	C12—C17—C16	120.4 (2)
N2—C8—C7	122.6 (3)	C12—C17—H17A	119.8
N2—C8—H8A	118.7	C16—C17—H17A	119.8
C7—C8—H8A	118.7	O3—C18—O4	123.4 (3)
C9—N2—C8	117.4 (2)	O3—C18—C16	123.2 (3)
N2—C9—C10	124.0 (3)	O4—C18—C16	113.4 (2)
N2—C9—H9A	118.0	C18—O4—H4C	112.6

### Hydrogen-bond geometry (Å, °)

**Table:**

*D*—H···*A*	*D*—H	H···*A*	*D*···*A*	*D*—H···*A*
O2—H2C···N1^i^	0.99	1.64	2.629 (3)	175
O4—H4C···N2^ii^	0.81	1.85	2.651 (3)	176

Symmetry codes: (i) −*x*, *y*+1/2, −*z*+1/2; (ii) −*x*+1, −*y*, −*z*+1.
